# Sinonasal Lymphoepithelial Carcinoma With Aggressive Orbital Invasion

**DOI:** 10.7759/cureus.31103

**Published:** 2022-11-04

**Authors:** Sruban Suparmaniam, Qi Zhe Ngoo, Wan-Hazabbah Wan Hitam, Patricia Ann Moknasing @ John

**Affiliations:** 1 Department of Ophthalmology and Visual Sciences, School of Medical Sciences, Health Campus, Universiti Sains Malaysia, Kota Bharu, MYS; 2 Hospital Universiti Sains Malaysia, Health Campus, Universiti Sains Malaysia, Kota Bharu, MYS

**Keywords:** proptosis, epstein-barr virus, ophthalmology, acute blindness, orbital, sinonasal tumor, lymphoepithelial carcinoma

## Abstract

Sinonasal lymphoepithelial carcinoma (LEC) is an extremely rare malignancy that shares some characteristics with nasopharyngeal carcinoma. In Asian populations, Epstein-Barr virus has been reported to be associated with LEC located outside of the nasopharynx. We report a rare case of sinonasal LEC with locoregional extension (brain and orbit). A 39-year-old Malay male initially presented with profound blurring of vision on the left eye (LE) and proptosis, followed by nasal symptoms of anosmia. Clinical examination revealed that the LE visual acuity was 6/36, with reduced optic nerve function with normal funduscopic findings, non-axial proptosis, and minimal limitation of extraocular movement. Subsequently, his vision worsened with perception of light in three days. Radioimaging studies showed soft tissue lesion at the ethmoid sinus with extensive local and intracranial extension. Microscopic analysis and immunohistochemistry confirmed the diagnosis of LEC. The patient was given induction chemotherapy followed by concurrent chemoradiotherapy with weekly intravenous cisplatin. Upon completing the fourth cycle of chemotherapy, the patient’s ocular symptoms and general conditions worsened. Repeated imaging showed worsening intracranial extension with cerebral and cerebellar edema, and the patient succumbed to death. Sinonasal LEC is a rare malignant tumor with little mention in the literature. This case was reported to highlight the importance of a high index of suspicion for acute ocular symptoms with mass.

## Introduction

The occurrence of malignant tumors of the paranasal sinus is uncommon [1]. Lymphoepithelial carcinoma (LEC) tends to be located in various sites in the head and neck; however, sinonasal involvement, especially in the ethmoid sinus, is extremely rare. According to the World Health Organization, sinonasal LEC is defined as “poorly differentiated squamous cell carcinoma and morphologically similar to nasopharyngeal carcinoma” with a strong association with Epstein-Barr virus (EBV) [2]. In most cases, sinonasal LEC presents with locally aggressive disease with or without regional lymph node metastases. It is found to be more common in Southeast Asia [3]. We report a rare case of LEC in the ethmoid sinus invading the orbit and brain, which initially presented with loss of vision and proptosis.

## Case presentation

**Figure 1:**
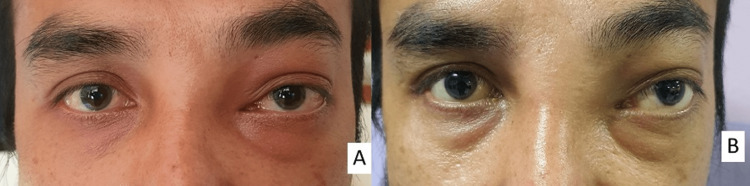
(A) Mild proptosis of the LE on admission. (B) Obvious non-axial proptosis after three days of admission. LE, left eye

A 39-year-old Malay male with underlying allergic rhinitis presented to the ophthalmology clinic with unilateral, painless progressive loss of vision for one week and proptosis for two months. The patient also had a history of recurrent headaches averaging one to two times per week for the past three months. He was a heavy smoker but then switched to e-cigarette (“vaping”) two years ago. There was no history of appetite loss or weight loss. Visual acuity on presentation was 6/36 in the left eye (LE) and 6/6 in the right eye (RE). There was a presence of relative afferent pupillary defect (RAPD) grade 2 in the LE with reduced red desaturation and light brightness. Both anterior segments were unremarkable. Fundoscopy showed normal optic disc and macula. The extraocular movement shows a limitation in LE lateral gaze and levoelevation with non-axial proptosis (inferolateral) (Hertel’s exophthalmometer at 112 mm: 15 mm in the RE and 18 mm in the LE). There was fullness of the left upper and lower lid extending towards the nasal bridge (Figure 1). Nasal endoscopy with uncinectomy and biopsy revealed a mass over the left lateral nasal wall occupying the entire maxillary antrum, medial meatus, roof of the maxillary sinus and ethmoid sinus, and easily bled. There was no lymphadenopathy.

**Figure 2:**
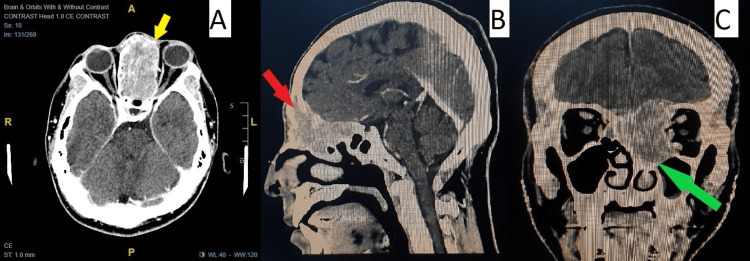
(A) Axial CECT (yellow arrow) shows a huge and highly aggressive mass from the ethmoid sinus extending to the left extraconal fat and stretching the left medial rectus muscle, abutting the intracanalicular part of the optic nerve. (B) Sagittal CECT (red arrow) shows destruction of the frontal bone, posterior wall of the frontal sinus, and cribriform plate with extension to the frontal sinus. Intracranial extension to the anterior cranial fossa was also noted. (C) Coronal CECT (green arrow) shows mass extending to the sphenoid sinus, maxillary sinus, and nasal cavity till the left middle meatus with disruption of the nasal bone. CECT, contrasted enhanced computed tomography

A contrasted-enhanced computed tomography (CECT) scan of the orbit, brain, and paranasal sinus (Figure 2) revealed an aggressive soft tissue lesion measuring 5.6 x 3.4 x 4.6 cm at the ethmoid sinus. There was an extensive local extension of the frontal sinus and maxillary sinus with bony destruction toward the frontal bone up to the pterygopalatine fossa. There was also intracranial extension (anterior cranial fossa) and in the orbital region toward the left extraconal stretching medial rectus, abutting the intracanalicular part of the optic nerve.

Biopsy of the mass revealed LEC. Immunohistochemistry showed tumor cells that were positive for pancytokeratin (pan-CK), cytokeratin CK5/6, and p63 gene with approximately 50% Ki-67 proliferation index, and negative for Epstein Barr encoding region in situ hybridization (EBER ISH). A diagnosis of sinonasal LEC with locoregional extension (brain and orbit) was made. The LE visual acuity of the patient vision deteriorated further to perception of light within a few days of admission. The LE proptosis became more prominent with the limitation of eye movement. The patient also had a nasal obstruction and anosmia (Figure 1B), but the RE remained normal. The patient was planned for induction chemotherapy of intravenous carboplatin and paclitexal 200 mg/m^2^ followed by concurrent chemoradiotherapy (CCRT) with a dose of 70 Gy for 35 cycles for seven weeks and weekly intravenous cisplatin 40 mg/m^2^. However, after completing the fourth cycle of chemotherapy, the patient developed headaches, seizures, and altered consciousness. Repeated CECT shows worsening intracranial extension with the involvement of the optic nerve. There was also the presence of diffuse cerebral and cerebellar edema. Subsequently, the patient succumbed to death after a few days.

## Discussion

Sinonasal malignant neoplasm accounts for 0.1% of all neoplasms and 60% involving the maxillary sinus. It has a strong predilection for males, with a ratio of approximately 3:1 in the fifth to seventh decade of life [4]. Sinonasal LEC is rare, and around 40 documented cases have been reported so far [5]. LEC typically affects the nasopharynx, salivary glands, and larynx in the head and neck region [6]. In this case, the LEC originates from the ethmoid sinus, which is exceedingly rare in the literature. Over the years, numerous terms have been used to describe LEC in extrapharyngeal sites, such as lymphoepithelioma, lymphoepithelioma-like carcinoma, lymphoepithelial-like carcinoma, undifferentiated carcinoma of nasopharyngeal type, and undifferentiated carcinoma with lymphoid stroma. Recently, it was accepted as a distinct entity separate from nasopharyngeal carcinoma (NPC) by topography and clinical outcome but histopathologically similar [7]. A study conducted in China shows the ratio of LEC to NPC to be 1:564 [8]. With a higher incidence rate of sinonasal LEC in Southeast Asian countries where ethnicity and geographical factors play a significant role, there is also an association with certain human leukocyte antigen (HLA) types, the southern Chinese diet, and EBV [6]. Around 90% of LEC cases are strongly associated with EBV. EBER ISH is the methodology of choice, which yields high sensitivity of detection for EBV [7]. In our patient, it tested negative for EBV. Nevertheless, inconsistent association between EBV and LEC has been observed in different races, such as in the USA and Western Europe, which are usually negative [9]. The status of EBV in the LEC of the maxillary sinus has no importance in prognostic value [10]. However, the treatment response and survival rate for the association of this virus and LEC have not been determined [1].

The signs and symptoms differ depending on the type, location, and stage of the malignancy [11]. The occurrence rate of sinonasal LEC is higher at the nasal cavity compared to paranasal sinuses. Commonly, patients present with nasal obstruction, epistaxis, and facial pain. Tumor invasion into the olfactory bulb, frontal lobe, and possibly brain stem causes anosmia and ageusia, whereas orbital invasion can lead to proptosis and cranial nerve palsies [3]. Symptoms such as epiphora, blood-tinged tears, and epistaxis were reported in a rare case of LEC of the nasolacrimal duct [12]. In a comparison between sinonasal LEC and nasopharyngeal LEC, the frequency of cervical lymph node metastasis was smaller in the former, reported at around 15%. Orbital and intracranial invasions were seen in 31% and 15% cases, respectively [13]. In our case, the patient presented with ocular signs and symptoms prior to nasal symptoms. LEC from the maxillary sinus can be an aggressive tumor with local invasions to nerves and orbit [9]. Smoking is not a risk factor for LEC in nasopharyngeal sites [14]. However, the association between sinonasal LEC and smoking is yet to be established. In our case, the patient was a heavy smoker and switched to e-cigarettes. A study on e-cigarette and nasal epithelial cells shows changes in the KI-67 positive cells and pro-inflammatory CK [15]. Differential diagnoses for LEC include melanoma, lymphoma, and sinonasal undifferentiated carcinoma (SNUC). SNUC, which comes as a diagnosis of exclusion, is highly regarded as an aggressive tumor. Compared to LEC, microscopically, SNUC demonstrates high mitotic activity and necrosis with no association with EBV [3].

On standard radiography, sinonasal LEC shows a diffuse opacity of soft tissue density. CT scan of paranasal sinus shows a homogenous mass occupying the sinus cavity, which does not enhance even with contrast. The LEC cannot be distinguished from other sinonasal malignancies, particularly squamous cell carcinomas or lymphomas, and all these tumors are locally invasive with characteristics to metastases to retropharyngeal and cervical node [9]. In terms of LEC definitive diagnosis, histopathological and immunohistochemistry analysis are warranted [11]. Microscopically, LEC is surrounded by poorly differentiated malignant epithelial cells with prominent infiltration of lymphocytes and plasma cells of the stroma [16]. In this case, immunohistochemical staining shows positive for pan-CK, indicating it is epithelial in nature with squamous differentiation from CK 5/6. There was a strong positivity for the proliferative marker Ki-67. The tumor cells are immunoreactive for the p63 gene, which highly signifies squamous cell carcinoma. In half of LEC cases, 10-75% of the tumors consist of squamous cell carcinoma components [17].

There is no standard treatment guideline for sinonasal LEC due to the rarity of this tumor and its anatomical complexity with close proximity to vital structures [3]. Initially, surgery is the treatment of choice; however, radiotherapy should be considered even if there is involvement of lymph nodes because LEC is radiosensitive [9]. Chemotherapy is recommended for non-nasopharyngeal LEC patients with regional adenopathy. These patients are at high risk of distant metastasis [2]. It can be given as neoadjuvant, concurrent, or adjuvant to radiotherapy [9]. A few studies stated the treatment of choice based on advanced NPC in view of similarities of LEC and NPC. In Japan, alternating chemoradiotherapy is given as a treatment choice for locoregional advanced NPC to minimize toxicity. The regime consists of three courses of chemotherapy (5-fluorouracil and cisplastin) and two courses of radiotherapy. Higher survival rates and lower toxicities are seen compared to CCRT. This method is suggested for sinonasal LEC in view of the high curative effect and better quality of life [2]. Another study administered chemotherapy of docetaxel plus carboplatin [1]. A long-term CT scan follow-up is necessary for maxillary sinus LEC since the recurrence rate is as high as 25% [6]. Sinonasal LEC has a five-year survival rate of around 50% [2]. The mortality rate per 1,000 patients is higher in Asian/Pacific Islander population, followed by African American/Black [16]. Our patient had cerebral and cerebellar edema while ongoing CCRT, and he died 10 weeks after the initial presentation.

## Conclusions

In summary, we reported a case of an aggressive tumor of EBV-negative sinonasal LEC with locoregional extension (brain and orbit) that initially presented to us with ocular signs and symptoms. Due to LEC's rarity, diagnosis is based on histopathological examination with immunohistochemical studies, but definitive therapy is still not established. Our case report reminds us of the need to bear in mind that a harmless appearing swelling combined with ocular complaints could be a rare malignancy. This rare and challenging tumor needs a systematic clinical approach with vigilance, proper diagnostic tests, and appropriate treatment. A high index of suspicion among clinicians for early recognition and timely referral with further studies to guide appropriate management is required for the betterment of patients.
